# Prevention and control of ship-source pollution in the Arctic shipping routes: challenges and countermeasures

**DOI:** 10.1007/s11356-023-30817-w

**Published:** 2023-11-18

**Authors:** Cheng Zhang, Jia-qi Yang

**Affiliations:** China Institute of Boundary and Ocean Studies, Wuhan University, Wuhan, 430072 China

**Keywords:** Arctic shipping routes, Ship-source pollution, Environment protection

## Abstract

Ship-source pollution is one of the important contributors to marine environment pollution. Because the legal status of the Arctic shipping routes is not clear, there is a considerable degree of dispute in the application of the rules on the prevention and control of ship-source pollution. The increased melting of sea ice undermines the legal legitimacy of the “ice-covered areas” clause under the United Nations Convention on the Law of the Sea. The conflict between the application of the Polar Code and “ice-covered areas” will also reach an initial conclusion in the context of melting sea ice. However, the inadequacy of ship-source pollution rules in the Polar Code hampers its application, which has led to a negative impact on the more active role in the governance of pollution from Arctic shipping. For replying to the Challenges in the prevention of ship-source pollution in Arctic shipping routes, the relevant rules of the Polar Code need to be further improved, while a more binding HFO ban according to ship types needs to be applied. Therefore, a more important role in the future Arctic governance mechanism will be played by the enhanced enforcement of the Polar Code, meanwhile, the target for uniform international regulation of preventing and controlling ship-source pollution in Arctic shipping routes should be achieved.

## Introduction

The Atlantic and Pacific Oceans are connected by a network of routes known as the Arctic shipping routes. The Northwest Passage (hence referred to as “NWP”), the Northeast Passage (hereinafter referred to as “NEP”), and the Transpolar Sea Route are the three main Arctic shipping routes (Arctic Council 2009). Since their discovery and construction, the Arctic shipping routes have had a long history of 300 years. Even if the Age of Discovery is past, the dream of uncovering new shipping lanes endures. The UK has traditionally been a leader in the exploration of the NWP and Northeast Passages, motivated by the need to learn more about international markets (Guo 2009). Following Canada’s independence in 1880, the UK ceded its jurisdiction over the NWP to Canada. The Great Northern Expedition launched Russia’s investigation of the Northern Sea Route (hence referred to as the “NSR”) in the eighteenth century (Xu 2017). And although limited by the economic impact of the fall of the Soviet Union, Russia has finally established its sovereignty over the NSR. The deepening of global warming has not only facilitated human activities into an active period but also significantly enhanced the navigability of Arctic shipping routes. The increasing passible with unescorted icebreakers in Arctic Shipping Routes have raised new issues for Arctic routes’ governance. The warming trend of governance issues marked by climate change, environmental pollution, navigation safety, and oil pollution leakage is as irresistible as global warming. The impact of Arctic environmental changes caused by the pollution in the Arctic shipping routes will affect the entire northern hemisphere countries, which determines that the issue of Arctic shipping routes governance will no longer be a regional issue, but a part of global ocean governance issues.

This paper adopts the research methods of literature analysis, normative analysis, and comparative analysis to discuss the multiple challenges of pollution prevention in the Arctic shipping routes (hereinafter “ASR”). The scope of the Arctic shipping route discussed in this paper only includes the NWP and the NEP, and the Transpolar Sea Route is excluded for unnavigability. In Marine Environmental Protection, ship-source pollution causes far less damage than land-based pollution. A scientific study shows that 44% of human-caused marine pollution is from land-based sources, 33% from atmospheric sources, and only 12% from ships (GESAMP 1990). However, this situation is different in Arctic shipping routes. Due to geographical location and ecological environment, human land activities are far away from the Arctic shipping routes. Ship source pollution has become one of the most important factors in pollution prevention and control of the Arctic shipping routes. The vessel’s construction, design, equipment, and manning directly control the pollution prevention. This article (Allen 2014) is divided into four parts, including the introduction, status quo of legislation in ASR, challenges enumeration, and coping strategy. We pay close attention to adopting pollution prevention mechanisms in Arctic shipping routes and amplify further consideration of rules supply in Arctic pollution prevention.

## Status quo of prevention and control of ship-source pollution in the Arctic shipping routes

In this section, governance subjects, governance rules, and governance objectives of ship-source pollution prevention and control in ASR will be reviewed.

### The participants: diversified prevention and control subjects

The responsibility to reduce and eliminate the threat to safeguard the pristine Arctic environment is within the maritime governance’s ability and authority. Now subjects of ASR governance span international organizations, forums, and coastal states, demonstrating a diversified trend of participation (Dalaklis et al. 2023). The International Maritime Organization (hereinafter “IMO”) which specializes in regulating ship affairs, the Arctic Council, the Climate and Clean Air Coalition to Reduce Short-Lived Climate Pollutants (hereinafter CCAC), as well as coastal states especially for Canada and Russia consisting of the diversified governing bodies.

The International Maritime Organization (IMO) may not have the same expertise in arctic issues as the Arctic Council, but it nonetheless has a significant impact on the prevention and management of ship-source pollution for its large number of signatories. IMO is given further authority with the initiation and implementation of the International Code for Ships Operating in Polar Waters (hereafter “Polar Code”). The Arctic Council, an international forum headed by eight Arctic nations, is without a doubt one of the most significant organizations in preventing and controlling ship-source pollution in ASR outside of the IMO. The Arctic Council is essential to Arctic governance, particularly for the environment preservation. In recent years, the Council has issued three legally binding international agreements that have greatly enhanced its governance influence and effectiveness, particularly in the areas of scientific cooperation and information sharing. The Arctic Council is experiencing operational challenges as a result of the Russia-Ukrainian conflict, but the impact has been somewhat mitigated by the low political sensitivity of environmental matters. After Finland’s initiation of the Arctic Environmental Protection Strategy (hereinafter “AEPS”), the Protection of the Arctic Marine Environment (hereinafter “PAME”) working group came into being. PAME joined the Arctic Council’s four working groups and played a significant role in the organization’s efforts to reduce and stop pollution from ships. PAME has conducted research on both established and uncharted areas of ship-source pollution, particularly in the ASR for wastewater discharge, fuel use, black carbon emission, and underwater noise pollution. Arctic Ship Traffic Data System (ASTD), aiming at collecting ship data in ASR, is also headed by PAME. The Arctic Council receives all the studies, which will serve as the foundation for new laws. PAME could be considered the cornerstone of Arctic Council ship-source pollution governance. The agreements, declarations, and frameworks reached at the Arctic Council ministerial meeting have made outstanding contributions to regularization and technical advancement in ASR. These agreements, declarations, and frameworks have formed a fixed declaration and reporting system, respectively playing a positive role in examining current issues and providing expert group recommendations in the field of Arctic shipping.

As for Russia and Canada, countries who are in the coastal of ASR, as well as the administrators of ASR in actuality, are authorized by the United Nations Convention on the Law of the Sea (hereinafter “UNCLOS”). Although both Russia and Canada try hard to “internalize” NSR and NWP, the international community never pays it. The United States and European Union contested Canada’s internalization claim, considering NWP is “an international strait, open to use by all states” (Thorsell and Leschine 2016).

ASR can efficiently sidestep any potential legal issues brought on by the ambiguous legal status of those routes by using Article 234’s “ice-covered areas” as their sole legal foundation. Under Article 234, Russia and Canada are empowered to adopt and enforce non-discriminatory laws and regulations for the prevention, reduction, and control of marine pollution from vessels in specific areas.^1^ It is worth noting that this right encompasses both legislative competence and law-enforcement power. Canada Shipping Act (hereinafter “CSA”) (2001) prohibits the discharge of oil into Canadian Waters, including the internalized Arctic Water. Penalties for violation of it are up to a $1,000,000 fine or 18 months in prison (Thorsell and Leschine 2016).

### Rules: the interwoven construction of the law of the sea and maritime law

The essential way to prevent, reduce, and control ship-source pollution in ASR is to establish targeted mandatory rules. In a broad sense, rules ensuring navigation safety are part of ship-source pollution prevention. Since collisions are another event that can cause pollution from ships. That is part of the reason why most laws and guidelines pertaining to safe sailing are obligatory. There are now “soft laws” and “hard laws” governing the prevention of pollution from ships. For example, the rules outlined in the Polar Code include both required and recommended provisions. The Law of the Sea and Maritime Law are both included in these regulations, which is their most notable aspect.

Article 234 “ice-covered area” authorizes coastal governments (particularly Russia and Canada) to legislate and implement rules and regulations inside the exclusive economic zone (hereafter “EEZ”). As mentioned before, such authorization constructs the foundation of justification in international law. Things changed when the Polar Code was introduced. Polar Code is not a stand-alone convention under IMO but in the form of three conventions’ amendments. International Convention for the Prevention of Pollution from Ships (hereinafter “MARPOL”), along with the International Convention for Safety of Life at Sea (hereinafter “SOLAS”) and International Convention on Standards of Training, Certification and Watchkeeping for Seafarers (hereinafter “STCW”) are the core conventions targeting marine pollution governance under IMO. The Polar Code was endowed mandatory as their amendments. Both Russia and Canada are parties to these three conventions. “Pacta sunt servanda” is the most basic principle in international law and was also written in Article 26 of the Vienna Convention on the Law of Treaties 1969. Russia and Canada do have obligations under IMO’s three conventions, both of them updated their unilateral legislation to cope with the Polar Code, especially for Canada (Kristin 2019). Despite their extensive efforts to adapt the Polar Code, there are still laws and regulations that transgress. Article 234 is more like a connection point between the Law of the Sea and Maritime Law, obligations under them are somewhat in conflict now. In the older draft of the Polar Code, it is noted that the Polar Code should not prejudice the development of UNCLOS, but such formulation has been changed finally (Thorén 2014).

Besides conventions mentioned earlier, 1990 International Convention on Oil Pollution Prevention, Response and Cooperation (hereinafter “OPRC”), 2001 International Convention for the Control of Harmful Anti-fouling Systems on Ships, as well as International Convention on the Control and Management of Ships’ Ballast Water and Sediments are also rules related.

### Goals: balance between environmental protection and economy utilization

ASR have great shipping development potential. Both coastal states and shipping companies can benefit more from extensive opening and commercial use (Li and Deng 2021). Such potential attracts interest on a global scale. Compared to the conventional Asia-Europe line via the Suez Canal, NSR can reduce the distance between Rotterdam in the Netherlands and Yokohama in Japan by more than 40% (Hong 2020). Additionally, the shortening will result in lower fuel and labor costs. As a result of the economic advantages, more ships are drawn to use the ASR. Unquestionably, incremental ships provide financial support for environmental governance. However, it also adds a significant burden on ship source pollution prevention and management. The major objective of preventing ship-source pollution is to find a balance between these two.

As a low-cost transportation, sea freight carries a huge share in goods transportation of international trade. Traditional shipping routes like the Suez Canal are already at capacity. ASR are the shortest from Europe to Asia, which means it can considerably take the load off traditional line. Experts predict that Arctic shipping will account for 2% of global shipping by 2030 and 5% by 2050 (Corbett et al. 2010). In March 2020, PAME released a report titled “The Increase in Arctic Shipping 2013–2019.” The report shows that ship volume in ASR increased rapidly during the year 2013 to the year 2019 (Arctic Council 2020). But due to the Arctic’s unique ecological environment, more stringent, appropriate, and delicate regulations are required to minimize ship-source pollution as more ships transit via ASR. The Polar Code makes an imperfect attempt to strike a balance between environmental protection and economic openness. Domestic laws in coastal states are currently quite far from achieving that objective. Currently, NSR and NWP are actually administered by Russia and Canada. These two nations exhibit divergent views toward environmental conservation and the economic development of the Arctic maritime routes as a result of their diverse political and economic environments. And these disparities have not been bridged after the enactment of the Polar Code (Bartenstein et al. 2022). Russia views the ASR as one of the key areas for economic growth in the future due to the long-term economic restrictions imposed by Western nations. Besides, NSR are more navigable compared with NWP. Therefore, rules and regulations in Russia are biased to economic development and the opening of NSR. Regulations related to pollution prevention are relatively relaxed. Canada has established stricter pollution prevention and control laws in light of the NWP’s actual situation. Prevention of pollution is the main focus of Canada’s unilateral regulations governing the ASR.

Russia’s and Canada’s different legislation tendencies are far from the balance goal. However, the shipping community’s attitude toward ASR is an even greater obstacle to its economic use. For several countries, “environmentally correct” is more like a kind of political endorsement. The Arctic Shipping Enterprise Pledge was introduced in October 2019 by the non-profit organization Ocean Conservancy and some of the biggest US businesses. Leaders in the shipping industry, including Evergreen and Sealand, have joined the voluntary joint endeavor. The pledge intends to bring together numerous businesses and shipping behemoths in opposition to the usage of ASR. Since it might be the most effective approach to stop pollution caused by ships. This pledge undoubtedly puts the economic utilization of the ASR at odds with environmental protection, let alone the balance goal. Moreover, NEP is the only Arctic shipping route that can now meet the demand for large-scale commercial navigation, but the NEP’s transportation capacity is severely constrained by the NEP’s deteriorating port infrastructure. The distinct magnetic storm phenomenon in the Arctic region and the lack of coverage of the International Maritime Satellite System significantly increases the insecurity of ASR. The aforementioned factors have become relevant considerations for international shipping companies to assess their investment costs. Therefore, allowing major international shipping companies to abandon the lucrative benefits of using existing international routes and instead invest high costs in developing ice-breaking technologies suitable for navigation in ice regions, while also bearing extremely difficult to control ship navigation pollution risks and predictable public opinion pressure, is obviously a move for major international shipping giants that are focused on practical benefits.

## Challenges: inadequacy rules and regulations in the ship-source prevention

Since the Polar Code came into force, the prevention and control system of ship source pollution in ASR has been improved by combining the law of the sea with the maritime law. However, the Polar Code, a brand-new rule that went into force in 2017, has a considerable degree of legislative gap. This is also one of the deep motivations for the disagreement between Canada and Russia over the observance of the Polar Code and the UNCLOS. This section will examine the lack of pertinent regulations in ship-source pollution prevention and control.

### Polar Code does not cover fishing vessels

The Agreement to Prevent Unregulated High Seas Fisheries in the Central Arctic Ocean enters into force in 2021. The Agreement has made the presence of fishing vessels in the Central Arctic Ocean impossible. But this is not the case for the entire Arctic Ocean. Only the Central Arctic Ocean, where there is permanent sea ice, is subject to the fishing prohibition. Therefore, natural conditions were the original constraint on commercial fishing in this area. The Agreement to Prevent Unregulated High Seas Fisheries in the Central Arctic Ocean only prohibits fishing where is not available, hence intensive fishing areas are not included.

According to PAME “the increase in Arctic shipping 2013–2019”, the volume of ships in ASR increased 25% from 2013 to 2019. And 41% are fishing vessels entering Arctic Waters defined by the Polar Code in 2019 (Arctic Council 2020). According to the data in the research, fishing vessels make up a sizable portion of the entire fleet. The majority of ships that are found in ASR are, quite literally, fishing vessels. Fishing vessels are not covered by the Polar Code, though. Because SOLAS and MARPOL are a component of the Polar Code, general SOLAS and MARPOL requirements also apply to the Polar Code. Regulation 3 of SOLAS shows that conventions do not apply to fishing vessels. It leads to an unreasonable situation in ASR. The safety of fishing vessels in Polar is hard to guarantee. Although the Polar Code sets high technical requirements for ship construction for ships sailing in polar waters, generally speaking, it is difficult for fishing vessels to meet the above technical standards for ship construction. The Polar Code should particularly define some necessary safety criteria for fishing vessels in order to guarantee dual safety in navigation and environmental dimensions, given the existing number of fishing vessels using ASR.

### Default of mandatory HFO ban

The legislative arrangements of the Polar Code attach much more importance to navigation safety, while the environmental protection rules are deficient in quantity and quality.

**Fig. 1:**
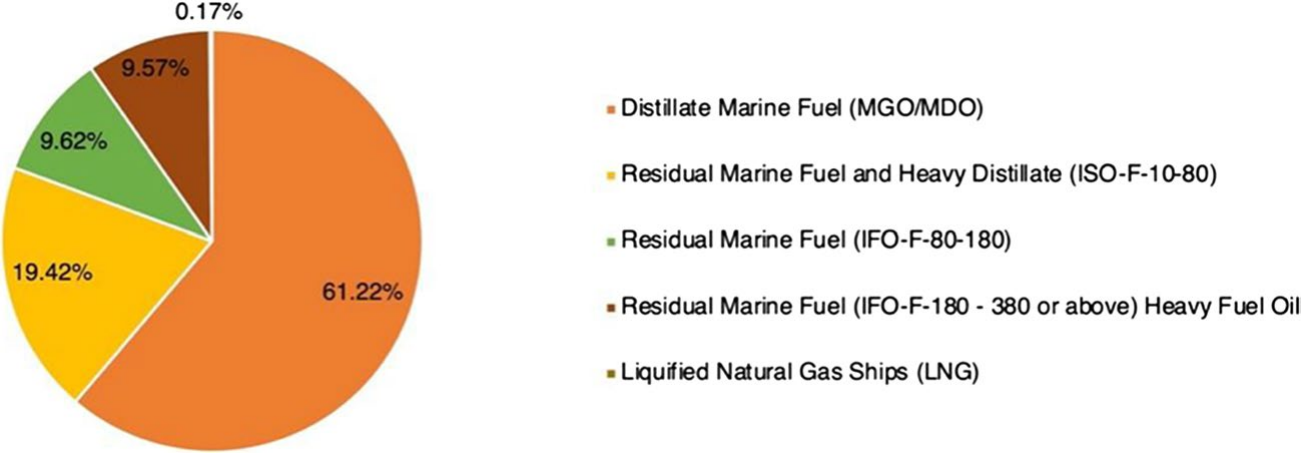
Numbers of ships using different types of fuels in arctic waters defined in Polar Code (2019). Data is collected from the Arctic Council (2020)

The use and carriage of HFO were outright prohibited in Antarctic Waters early in 2011. The low price of HFO has made it one of the most popular fuels in unrestricted waters, including ASR. By 2015, HFO had accounted for 57% of Arctic Shipping (Comer et al. 2017a, b). As a kind of fuel with high toxicity and viscosity, HFO will emit a lot of sulfur oxide and black carbon after combustion, which is one of the main sources of ship source pollution. Considering the characteristics of HFO, leakage of HFO will result in a serious ecological catastrophe. Consequently, research on HFO governance has been ongoing for several years. The PAME Working Group under the Arctic Council has produced a multistage report on the use of HFO in Arctic waters since 2011, so as IMO. However, the emphasis on HFO did not continue in the Polar Code. The Polar Code did not ban HFO initially. Figure 1 shows the use of HFO in ASR in 2019. It is clear that HFO will continue to be the primary fuel used in ASR through 2019.

Things changed in 2020 nevertheless. Since January 2020, IMO has mandated the use of very low sulfur fuel oil (hereinafter “VLSFO”) with a sulfur content of less than 0.5%. IMO 2020a, b sulfur cuts seem to provide a buffer period for the eventual introduction of the HFO ban. The sub-committee on Pollution Prevention and Response under the IMO (hereinafter referred to as “PPR”) was held in London in February 2020. The meeting agreed to amend Annex I of the MARPOL Convention in order to reconcile the different countries’ interests upon the HFO ban. The draft amendment clearly pointed out that ships will be completely prohibited from using and carriage of HFO in Arctic Waters from July 1, 2024. Ships engaged in safety protection, search and rescue operations, or meeting the fuel oil tank protection requirements are exempt from the HFO ban. Additionally, ships registered in Arctic states will also be exempt. Ships meeting fuel oil tank protection requirements and Arctic States’ ships have a 5-year transition. The exemption ends in 2029. The long period and large scope of exemption made the so-called HFO ban ineffective (IMO 2020a). Before this deadline, 74% of ships are allowed to continue to use HFO. Thus, the risk has not been eliminated (Bai 2021). In addition, the ban on HFO in the Arctic only applies to Arctic waters within the scope of the Polar Code. However, a 2016 report by the PAME Working Group pointed out that most navigation accidents occur near the North Pole, which is not covered by the Polar Code (Bai and Chircop 2020).

### Rules for black carbon emission are still not mandatory

In terms of black carbon emission, the Arctic Council and the IMO’s methods are even less satisfactory. Black carbon, also known as black carbon aerosol, is amorphous carbon formed through incomplete combustion of fossil fuels, biofuel, and biomass. Black carbon has been noted for a long time as a potent climate forcer in atmospheric pollution (Ghosh and Rubly 2015). Although the emission volume compared with the world’s shipping is tiny (US Environmental Protection Agency 2012), the regional impact in the Arctic is especially noticeable since it darkens snow and ice, thus reducing the albedo effect (Yuan 2019).As a short-lived climate forcer, an immediate reduction of black carbon emissions could effectively curb Arctic warming in the next few decades (Bond et al. 2013).

The report “Black Carbon Emissions and Fuel Use in Global Shipping 2015” published by the International Council on Clean Transportation (hereinafter “ICCT”) revealed that the greenhouse effect of black carbon emissions at high latitudes (60°–90°N) is nearly five times that of low and medium latitudes (28°–60°N) (Comer et.al. 2017). However, since black carbon does not belong to the scope of Greenhouse Gas, it is not covered by the United Nations Framework Convention on Climate Change and its Kyoto Protocol, as well as the Paris Agreement. In 2009, the Executive Body of the Convention on Transnational Long-range Air Pollutants (hereinafter “CLRTAP”) introduced black carbon emissions into their research and paid special attention to the role black carbon plays in the Arctic.^2^ In the Gothenburg Protocol, whilst including black carbon emission provisions, it is still voluntary.^3^Furthermore, Arctic states such as Canada and Russia have not ratified the Protocol. It means that black carbon emission governance did not settle in CLRTAP. When MARPOL was amended to make the Polar Code mandatory, annex VI “prevention of air pollution from ships” was not revised. Therefore, black carbon emissions were not even mentioned in the Polar Code. The reasons are complex, and the opposition from the Arctic States is one of the most important factors. Various measures to reduce black carbon emissions are bound to increase shipping costs, which will greatly affect the lives of indigenous peoples in the Arctic. In February 2020, the IMO sub-committee on Pollution Prevention and Response (PPR 7) identified the three most appropriate black carbon measurement methods, light absorption filter smoke number, photo-acoustic spectroscopy, and laser-induced incandescence. However, whilst the measurements mentioned before can make an accruable investigation of black carbon emissions, IMO proposed the reduction of black carbon is still in an advisory form, with no mandatory.

It is also important to stress that the use of VLSFO had an unintended negative influence on black carbon emissions. Studies have shown that the high content of aromatic compounds in VLSFO will lead to higher levels of black carbon emissions. Although the actual situation of black carbon emissions from ASR still needs further monitoring and evaluation (IMO 2020b), the negative impact of black carbon emissions on Arctic warming and pollution should not be underestimated. How to balance the use of HFO and black carbon prevention and control in the process of innovation in the Polar Code requires IMO to propose more targeted institutional responses based on full demonstration.

## Strategies for challenges in the prevention of ship-source pollution in the Arctic shipping routes

The prevention and control of ship-source pollution is not only aimed at environmental protection to a certain extent. Uniform rules for the prevention and control of pollution from ships are also the basis for convenient maritime transportation and the result of the joint consideration of economic, environmental, political, and various factors. The introduction of the Polar Code provides the possibility of applying uniform ship-source pollution prevention and control rules for the ASR. However, whilst all the Arctic States are parties to the Polar Code, the application of unilateral rules from coastal states of the ASR constitutes the major obstacle of applying uniform rules. Thus, how to dilute the divergence in the application of the rule is important but difficult.

### Improving relevant rules in the Polar Code

The large exemption scope of the HFO ban and lack of mandatory rules of black carbon emission in ASR has caused considerable obstacles to the governance of ship-source pollution. Moreover, it has been one of the reasons for Russia and Canada to implement their unilateral rules. The inadequacy of the environmental protection rules of the Polar Code are “legitimate excuse” for applying more stringent unilateral rules, hindering the unification of pollution prevention standards seriously.

During the negotiation of the Polar Code, Canada, and Russia clearly showed their attitude towards the relationship between the Polar Code and their domestic unilateral legislation. Russia presented their intention literally, considering that their domestic law would take precedence over the application of the Polar Code.^4^ Both Countries’ attitudes have not changed since the Polar Code came into force. Russia and Canada revised their domestic legislation after the Polar Code came into force. But the revised rules still go beyond the Polar Code. For instance, emission standards in the Arctic Shipping Safety and Pollution Prevention Regulations (hereinafter “ASSPPR”), Canada’s domestic law for NWP, are more stringent than standards in the Polar Code. At the same time, the navigable area and time are restricted for specific types of ships. Furthermore, non-SOLAS ships must be equipped with pilots in specific areas.^5^

Considering all the circumstances, the improvement of the relevant rules in the Polar Code is not only necessary for the protection of the fragile ecological environment in the Arctic but also becomes the basis for the uniform application of the rules on preventing ship-source pollution. Safety rules for fishing vessels, a more effective ban on HFO, and regulations on black carbon emissions are the top priorities for the improvement of the Polar Code. The most likely development of the Polar Code in the future is to absorb the unilateral rules from Russia and Canada. The Polar Code consists of safety rules and pollution prevention rules. Rules under the two parts include both mandatory and advisory. Given the disobedience of Russia and Canada, incorporating reasonable rules from their domestic law seems to be an effective way to bridge the conflict between the Polar Code and Article 234 of UNCLOS.

### Promotion of fishing vessels’ navigation safety in Arctic water

As the Polar Code does not cover fishing vessels, then navigation safety of them is always under consideration. Revisiting all the international conventions or agreements involving vessels, their forceable are not as ideal as we looking forward to.

Maybe in view of the stagnation of the Cape Town Convention, the Polar Code waive to contain rules related to fishing vessels. On the one hand, the Cape Town Convention is expected to be forceable in the future; On the other hand, they are concern the inclusion of rules to safeguard fishing vessels will hinder the enforcement of the Polar Code. However, when the Cape Town Convention took effect is still unknown. In October 2019, a total of 48 states participated Ministerial conference in Spain and signed the Torremolinos Declaration to show their determination and ensure that the Cape Town Convention will come into force by October 11, 2022. Then, this plan fell apart, unexpected but reasonable. In the absence of the Cape Town Agreement, perhaps the IMO should take the lead in developing general rules for fishing vessels in Polar Water to safe both navigation and the environment. In September 2023, the IMO Maritime Safety Committee approved the Guidelines for safety measures for fishing vessels of 24 m in length and over-operating in polar waters (MSC.1/Circ.1641). But these new guidelines are not forceable as before.

Moreover, the Cape Town Convention and the new Guidelines only include vessels over 24 m, which means that fishing vessels that do not satisfy this standard are still not covered. We still expect more comprehensive and enforceable rules to protect the Arctic environment. Legislation tailored for Polar Waters could represent a fresh effort towards establishing international regulations for fishing vessels, potentially expediting the implementation of the Cape Town Convention.

### Applying more binding HFO ban according to ship types

The emission of black carbon from ships in sea transportation mainly comes from the combustion of HFO. In 2015, according to IMO definition, more than 2,000 ships sailing in the Arctic emitted 193 tons of black carbon, 68% of which came from the HFO (Yuan 2019). The existing HFO ban is based on ship structure and ship registration, resulting in a massive exemption of up to 74% of ships by 2029.

To issue a ban with a huge exemption upon HFO is the result of balancing multi-interests. A more effective HFO ban may have a great impact on indigenous in the Arctic and the transportation of Arctic energy resources. Obviously, the arrangement upon HFO is not satisfactory enough. On the basis of balancing different interests, the HFO ban can go further. The report “Black Carbon Emissions and Fuel Use in Global Shipping 2015” published by ICCT shows that container ships, bulk carriers, and tankers together accounted for 60% of black carbon emissions throughout 2015, occupying 30% of global ships and 81% of deadweight tonnage. Meanwhile, cruise ships make up just 1% of the global fleet but contribute 6% of black carbon emissions (Comer et al. 2017a, b). If ships such as cruise ships are classified to apply more stringent HFO ban regardless of their registration, black carbon emissions can be effectively reduced. In 2018, an analysis on influences HFO ban brings to cruise shipping took MS Rotterdam, which IMO registration number is 9122552, as an object to initiate a case study. The study aimed at finding the impact of HFO ban on fuel costs and ticket prices of cruise ships. The study found that the HFO ban would increase fuel costs for ships by 15–25% per voyage, but it also noted that the impact on Arctic commercial cruise traffic would be minimal because the increase in parts would be spread over 1400 tickets. And if operators share the cost increase with passengers, the ticket increase will be much more minimal (Abbasov and Gilliam 2018). Furthermore, limiting the exemption within internal waters and territorial seas may also contribute to reducing 70% of HFO carriage and 75% of HFO use (Comer et al. 2020).

For the sake of ship owners’ economic cost-effectiveness and competitiveness of ASR, IMO may consider giving further guidance for energy use to ship owners, coping with more binding black carbon emission restrictions. The wind propulsion system may be a better choice to save cost and clean environment, but studies have shown that such a combination will take up more open deck and then take over cargo’s volume. Considering all these things, lower tax rates seem to mitigate negative influences. However, taxation reduction needs further communication with the surrounding Arctic Nations (Kong et al. 2021).

## Conclusion

There are ways to prevent ship-source pollution in ASR but need mandatory rules to support. As a new navigational regulation especially applicable to polar waters, the Polar Code still has great room for improvement. Such improvement depends more on advances in Marine technology. At the same time, the progress of Marine technology will enhance the autonomous navigation ability of ships, which will also reduce the dependence of ships on safety rules. What is more, it can bridge the conflict between the unilateral legislation of coastal states and the Polar Code to a certain degree. The application of Article 234 might be even more controversial as the sea ice melting intensifies. The Polar Code will be more applicable considering the legal justification. As the main legal link between maritime law and maritime law, the international community expects and needs a more consummate Polar Code. With the improvement of the shipping capacity of the ASR, Polar Code seems to face more challenges. Thus, further research and update is of great necessity for IMO. In the above-mentioned innovation process of promoting the content and effectiveness of the Polar Code, the IMO should also take the “two-pronged approach” of navigation safety and environmental safety as the main tone, and formulate more complete rules to strengthen the response capacity to Arctic ship-source pollution, in order to strengthen the governance role of the Polar Code as an important link in the Arctic governance mechanism while bridging differences between the participating members of Arctic affairs, improve its governance capacity and applicable effectiveness for the legal regulation of ASR. IMO should keep a watchful eye on the communication and consensus of the Arctic Nations to advance the Polar Code.

## Data Availability

No new data is available for this article.

## Footnotes

1 UNCLOS,234: coastal states have the right to adopt and enforce non-discriminatory laws and regulations for the prevention, reduction and control of marine pollution from vessels in ice-covered areas within the limits of the exclusive economic zone, where particularly severe climatic conditions and the presence of ice covering such areas for most of the year create obstructions or exceptional hazards to navigation, and pollution of the marine environment could cause major harm to or irreversible disturbance of the ecological balance. Such laws and regulations shall have due regard to navigation and the protection and preservation of the marine environment based on the best available scientific evidence.

2 Executive Body of the CLRTAP, Decision 2009/5 Establishment of an Ad Hoc Expert Group on black carbon ECE/EB.AIR/99/ADD.1.

3 Gothenburg Protocol art 3(6).

4 Canada, Proposed Framework for the Code of Ships Operating in Polar Waters, IMO Doc. DE 53/18/2, 20 November 2009, at 7, paragraph 2.11 and Russian Federation, Procedure of Accounting for National Regulations, IMO Doc. DE 55/12/23, 1 February 2011, paragraph 5.

5 Arctic Shipping Pollution Prevention Regulations ASSPPR Sect. 8 (1) and schedule 1; Sect. 10.
